# Tracing the Thermal History of Seafood Products through Lysophospholipid Analysis by Hydrophilic Interaction Liquid Chromatography–Electrospray Ionization Fourier Transform Mass Spectrometry

**DOI:** 10.3390/molecules23092212

**Published:** 2018-08-31

**Authors:** Ilario Losito, Laura Facchini, Rosa Catucci, Cosima Damiana Calvano, Tommaso R. I. Cataldi, Francesco Palmisano

**Affiliations:** 1 Dipartimento di Chimica, Università degli Studi di Bari “Aldo Moro”, Via E. Orabona 4, 70126 Bari, Italy; facchini.laura@gmail.com (L.F.); catucci.rosa@tiscali.it (R.C.); cosimadamiana.calvano@uniba.it (C.D.C.); tommaso.cataldi@uniba.it (T.R.I.C.); francesco.palmisano@uniba.it (F.P.); 2 Centro Interdipartimentale SMART, Università degli Studi di Bari “Aldo Moro”, Via E. Orabona 4, 70126 Bari, Italy

**Keywords:** lysophospholipids, seafood products, thermal treatments, hydrophilic interaction liquid chromatography, high resolution mass spectrometry

## Abstract

Low temperature treatments commonly applied to seafood products have been shown to influence their phospholipid (PL) profile through enzymatic hydrolysis. In the present study, the generation of lysophospholipids (LPL) resulting from this process was systematically investigated for selected, commercially relevant seafood products, namely oysters, clams, octopuses, and shrimps. These products were subjected to thermal treatments like refrigeration or freezing after being purchased as fresh, defrozen, or frozen products depending on the case. The coupling between hydrophilic interaction liquid chromatography (HILIC) and electrospray ionization with high resolution/accuracy Fourier transform mass spectrometry (ESI-FTMS) was exploited to evaluate the PL profile of the cited products, especially the incidence of LPL related to the two main PL classes of seafood products—phosphatidylcholines (PC) and phosphatidylethanolamines (PE)—in the lipid extracts. The lyso forms of PE (LPE) were found to be generally more sensitive than those of PC (LPC) to thermal treatments, usually exhibiting a significant increase upon prolonged refrigeration at 4 °C in all types of investigated products except European flat oysters. Moreover, the distinction between fresh and frozen or defrozen products could be achieved in the case of octopuses and shrimps, respectively.

## 1. Introduction

The consideration of seafood products as a fundamental component of a healthy diet has increased constantly in the last three decades due to the beneficial effects related to their macro- and micronutrients [1,2,3,4]. The consequent growth in demand on a worldwide scale has led to a rapid globalization of the market, with an increasing need for long-range transportation and long-term storage of these products [5]. Recent statistics show the prevalence of Asian countries (especially China) among the most relevant countries in terms of seafood production based on capture or aquaculture, and remarkable trade flows occur between Asia and traditionally important markets for seafood, such as North American and European countries [5]. 

The steady increase observed in the incidence of frozen seafood products in these markets [5] is a direct consequence of this commercial trend. Indeed, freezing is the thermal treatment performed—usually soon after capture (on board of fishing vessels)—on fishes, cephalopods (i.e., octopuses, squids, and cuttlefish), and crustaceans (shrimps and prawns), which are subjected to intercontinental transportation and subsequent long-term storage with the aim of preserving them from oxidative deterioration [6]. A preliminary treatment at freezing temperatures—known as “glazing” [7]—is also applied in the case of a specific type of bivalve mollusk—mussels, especially those of *Mytilus chilensis* sp.—which are farmed along the coasts of Chile and are then delivered as glazed product to North America and Europe. Less severe thermal treatments, namely refrigeration at a few °C or chilling on ice, are usually indicated by current legislation for seafood products that are commercialized fresh or defrozen. Refrigeration is also applied in the case of shellfish (mussels, clams, oysters, etc.) sold as live products (see, for example, Annexes VII and VIII of the European Regulation 853/2004). 

Unfortunately, all the cited treatments may result in significant alterations of the lipid component of seafood products, which is particularly important in terms of nutritional and health benefits [1,2,3]. More specifically, as emphasized by several investigations performed in the last two decades, seafood lipids can undergo oxidative and/or hydrolytic reactions because of low temperature thermal treatments, especially freezing [6,8,9,10,11,12,13,14,15]. In this research context, the coupling of hydrophilic interaction liquid chromatography (HILIC) with high resolution/accuracy Fourier transform mass spectrometry (FTMS) based on electrospray ionization (ESI) has been previously successfully exploited in our laboratories to emphasize the increase in lysophospholipids occurring in gilthead sea bream (*Sparus aurata*) fillets frozen at −20 °C [16]. More recently, the same analytical approach—benefiting from the well-known ability of HILIC to separate different phospholipid classes based on the type of polar head—has been exploited for an extended characterization of the profile of glycerophospholipids [17,18] and ceramide lipids [19] in commercial mussels of *Mytilus galloprovincialis* sp. (Mediterranean mussel). As a result, specific seasonal variations occurring in fresh mussels farmed in aquaculture plants, which are clearly related to changes in the sea temperature, were evidenced [17], and a remarkable increase in the incidence of lysoPL, especially LPE, was observed after freezing mussels at −15 °C [18]. A similar effect occurred in the case of mussels that did not remain alive after prolonged (eight days) refrigeration at 4 °C, whereas those still alive (which represented 100% of the lot for refrigeration times not longer than four days) exhibited an LPL incidence comparable to that of fresh/live mussels [18]. This scenario was confirmed by a careful characterization of fatty acids released in the mussel tissues/organs upon the same thermal treatments, which led to a significant increase in LPL [20]. Interestingly, despite the low temperature adopted in the last stage of processing, glazed mussels of *M. chilensis* sp. and cooked/frozen mussels of *M. galloprovincialis* sp. did not shown any appreciable increase in the incidence of LPL compared to fresh ones [18]. This apparently surprising outcome was explained by considering the heat-induced inactivation of endogenous phospholipases, which are the main enzymes responsible for PL hydrolysis that occur during low temperature treatments of seafood products.

Starting from the results described so far, an investigation based on HILIC–ESI-FTMS was recently undertaken to assess whether the variation of LPL incidence could also be exploited as a marker of low temperature treatments for other commercially relevant (at national or international level, according to the case) seafood products that are potentially characterized by a more diversified set of thermal treatments. Among the latter, bivalve mollusks, cephalopods, and crustaceans—usually obtained from commercial sources—were considered. In particular, oysters of *Ostrea edulis* sp. (European flat oyster) and *Crassostrea gigas* (Pacific or Japanese oyster) and clams of *Ruditapes philippinarum* sp. (Manila clam) were selected among bivalve mollusks. The latter are usually obtained through aquaculture and commercialized as a live product that can be stored only for a few days under refrigeration at a temperature of a few °C, according to the current European legislation (see European Regulation 853/2004). As for cephalopods, *Octopus vulgaris* (Common octopus) was selected as a representative worldwide-diffused octopus species that are usually commercialized as fresh, defrozen, or frozen product. Finally, two species of shrimps were considered among crustaceans. The first was *Parapaeneus longirostris* (Mediterranean pink shrimp), which are obtained by capture in the Mediterranean area and are available as fresh product on the European market. The second was *Metapenaeus monoceros* (speckled/brown shrimp, also known as Indo-pacific shrimp), which are captured in the Indian or Pacific oceans and are commercialized in European and North American markets as defrozen or frozen product. It is worth noting that although several studies have been already dedicated to the lipid fraction of some of the selected products—namely clams of *Ruditapes philippinarum* sp. [21,22,23], oysters of *Ostrea edulis* sp. and *Crassostrea gigas* [21,24], octopuses of *Octopus vulgaris* sp. [25,26], and shrimps of *Parapenaeus longirostris* sp. [27]—no systematic investigation has been reported so far on the evolution of their PL with thermal treatments.

Some of the seafood products selected for the present investigation offered an opportunity to evaluate the effect of a thermal treatment that has not been explored before in terms of its possible effects on the PL profile. Indeed, the sequence of freezing (usually occurring soon after capture on board of fishing vessels) and thawing (usually performed just before commercialization), which are typical of octopuses and shrimps commercialized as defrozen products, could be studied. The eventual differences in LPL incidence occurring between fresh, defrozen, and frozen products—along with the effects of prolonged refrigeration on the LPL abundance in live bivalve mollusks and in fresh or defrozen octopuses and shrimps—could thus be evaluated. The results arising from this extended study, which emphasizes the diversities in the evolution of the PL profile occurring in different products even if subjected to the same thermal history, will be described and discussed in the present paper.

## 2. Results

### 2.1. HILIC–ESI-FTMS Analysis of Phospholipids in Fresh Seafood Products

**Figure 1:**
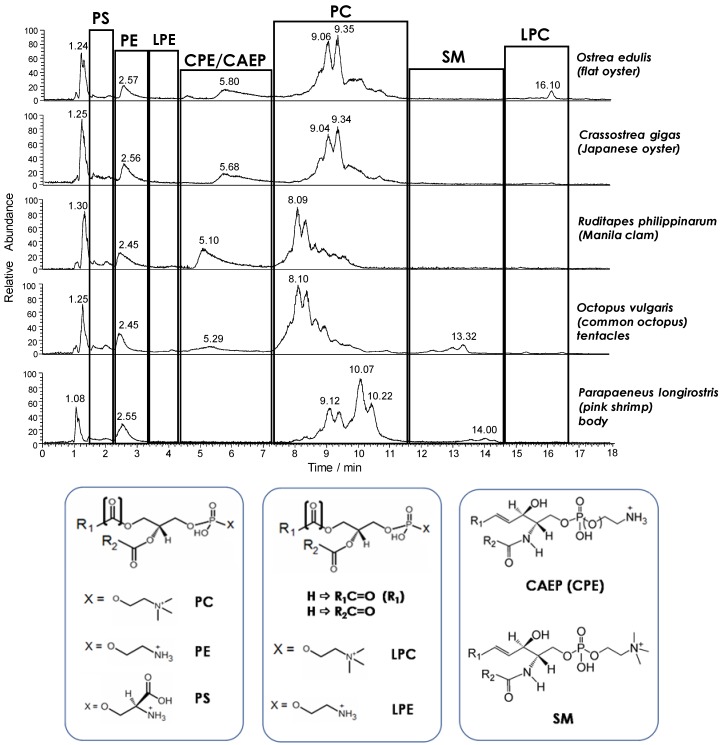
Comparison between hydrophilic interaction liquid chromatography–electrospray ionization(+)-Fourier transform mass spectrometry (HILIC–ESI(+)-FTMS) total ion current (TIC) chromatograms obtained for the lipid extracts of different seafood products, which were all purchased as fresh products and analyzed without further thermal treatments. The general structures of positive ions related to species belonging to the detected phospholipid (PL) classes are reported, with R_1_ and R_2_ representing saturated or unsaturated alkyl chains.

By analogy with previous investigations on mussel phospholipids [17,18,19], HILIC–ESI(+)-FTMS was adopted for the initial characterization of the main PL classes in the seafood products of interest, which were all examined as fresh products in the first step of the study. As expected, the total ion current (TIC) chromatogram resulting from HILIC separation provided a snapshot of the PL class distribution in each product (see Figure 1). The retention time window of each PL class was easily inferred from searches on the LipidMaps database based on accurate *m*/*z* ratios retrieved from FTMS spectra averaged along the window, as described in the Materials and methods section. As emphasized in Figure 1, besides glycerophospholipids such as PC, PE, and phosphatidylserines (PS), ceramide PLs were also detected. Notably, ceramide amino-ethyl-phosphonates (CAEP) and ceramide phosphoethanolamines (CPE) were only found in bivalve molluscs, like in the case of mussels [17,18,19]. On the other hand, sphingomyelins (SM) were found in lipid extracts of shrimps, whereas all the three types of ceramide lipids were detected in the extracts of octopuses, thus marking a definite evolution of the ceramide lipid component in the marine organisms under study. A further important feature of lipid profiles compared in Figure 1 was the generally low response observed for lysoPC (LPC) and lysoPE (LPE). This finding confirmed the very limited incidence of PL hydrolysis previously observed in fresh mussels [18]. Last but not least, variations occurring between the profiles of bands related to the same PL class in different products could be inferred from HILIC–ESI(+)-FTMS traces like those of Figure 1. The effect was very evident in the case of PC, representing the leading PL class in all seafood products, at least in terms of ESI-FTMS overall response. As apparent from Figure 1, several distinguishable—although not completely resolved—peaks were always observed in the retention time interval related to PC, leading to characteristic chromatographic profiles. The PC profiles related to the two types of oysters under analysis were rather similar, as expected, with major peaks located between 8.5 and 9.5 min, whereas peaks eluting between 7.5 and 9 min prevailed in the case of clams and octopuses. It is worth noting that the TIC trace related to the lipid extract of octopus mantle, not shown in Figure 1, was almost identical to the one related to the tentacle extract. The PC profile observed for shrimps was different from all the others, with two groups of peaks located around 9 and 10 min, respectively. 

**Figure 2:**
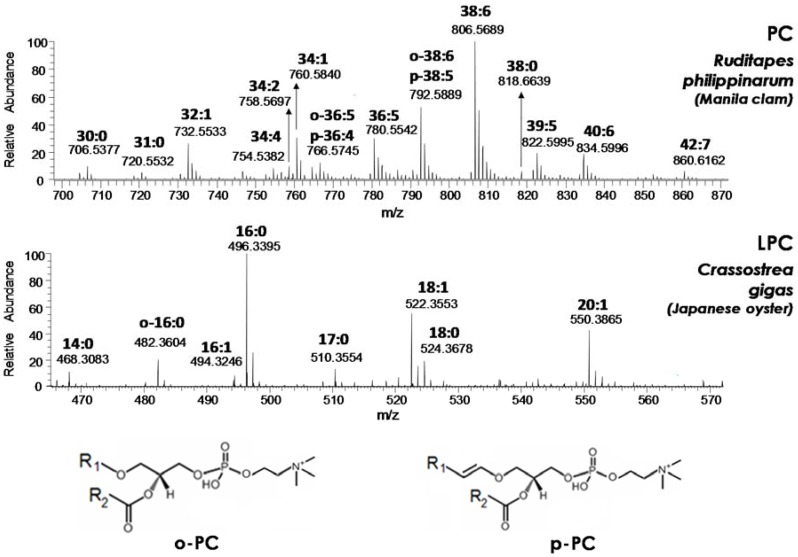
Averaged ESI(+)-FTMS spectra related to phosphatidylcholines (PC) and lysophosphatidylcholines (LPC) classes obtained, respectively, from the HILIC–ESI(+)-FTMS analysis of lipid extracts of Manila clams (*R. philippinarum*) and Japanese oysters (*C. gigas*). Spectral averaging was performed in the following retention time intervals: 7.3–11.6 min for PC and 14.6–16.7 min for LPC (see Figure 1). The molecular structures of plasmanyl-PC (o-PC) and plasmenyl-PC (p-PC) are also reported.

These variations, which clearly reflect the biological diversity of the examined products, could be evaluated in more detail from a molecular point of view by considering the ESI-FTMS spectra averaged in retention times intervals related to specific PL classes. In the present study, the attention was focused on the two main types of PL—PC and PE—and on their lyso forms, since, by analogy with mussels [18], the latter were expected to be increased after at least part of the performed low temperature treatments. As an example, the ESI(+)-FTMS spectra retrieved for PC and LPC classes after the analysis of lipid extracts obtained from two of the seafood products under investigation—Manila clams and Japanese oysters (analyzed as fresh products, soon after purchase)—are reported in Figure 2. Cross-comparisons of ESI(+)-FTMS spectra obtained for the four PL classes of interest—PC, LPC, PE, and LPE—from all seafood products under investigation have been reported in Figures S1–S4 of the Supplementary Materials, respectively.

The overall composition related to the side chain(s) characteristic of each species detected in a PL class, as indicated by the conventional C:D notation (where C represents the total number of carbon atoms of side chains linked to glycerol and D the total number of C=C bonds), could be obtained from spectra like those shown in Figure 2 and Figures S1–S4. In detail, accurate *m*/*z* ratios related to the M+0 isotopologue of each species could be retrieved and used as input for a search on the LipidMaps database after setting a ±0.005 mass tolerance. This tolerance is higher than the actual accuracy (around ±0.001 units) available on *m*/*z* ratios provided by the Q-Exactive spectrometer when operated under high mass resolution/accuracy conditions, as in the present case. Nonetheless, the choice of a higher tolerance was aimed at compensating the worsening of accuracy occurring for low S/N signals. In any case, signals related to M+0 isotopologues and having a relative abundance higher than 5% were labeled with the corresponding overall chain composition in Figure 2 and Figures S1–S4. In accordance with the generally accepted nomenclature, PL characterized by the presence of an alkyl chain—a side chain linked to glycerol through an ether, instead of an ester, bond—were labeled with the C:D notation preceded by o- or p-. The two letters were related to plasmanyl-PL (bearing C=C bonds on the alkyl chain but not in the proximity of the ether bond) and plasmenyl-PL (in which a C=C bond is certainly adjacent to the ether O atom), respectively, as exemplified in Figure 2 for o-PC and p-PC. It is worth noting that, as a convention, the C=C bond adjacent to the ether O atom is not accounted for in the C:D notation for p-PL; thus, PLs labeled as o-C:D and p-C:(D-1) are isobaric. MS/MS can provide diagnostic information to distinguish such isobaric PLs (see, for example, Reference [17]), yet this level of structural elucidation was beyond the goals of the present study. Consequently, both the possible assignments were reported in spectra of Figure 2 and Figures S1–S4 when ions with *m*/*z* ratios compatible with the presence of isobaric plasmanyl/plasmenyl species were detected in ESI(+)-FTMS spectra.

As apparent from Figure 2, plasma(e)nyl PLs had a limited incidence, at least in terms of number of detected species, in the case of Manila clams (Ruditapes philippinarum) PC, which were dominated by diacylic species. This finding was confirmed in all the other seafood products, with only one further plasma(e)nyl-PC (o-34:1/p-34:0) detected in the lipid extract of octopus tentacle (see Figure S1). Not surprisingly for marine organisms, most PC of the analyzed products (see Figure 2 and Figure S1) exhibited a remarkable degree of unsaturation in their side chains, with up to seven C=C bonds, whereas the abundance of saturated (i.e., 30:0, 31:0 and 38:0) or monounsaturated (i.e., 32:1, 33:1 and 34:1) species was generally low. With regard to the total number of carbon atoms, even values (the entire series from 30 to 42) prevailed, as expected; yet, odd values were not completely absent (see labels 31:0, 33:1, 39:5 and 39:6 in Figure 2 and Figure S1). Note that relative abundances observed in spectra related to a specific PL class—like those of Figure 2 and Figures S1–S4—can be reasonably considered as corresponding to relative concentrations. Indeed, a previous study performed on PL mixtures and based on HILIC–ESI-MS showed ESI yields for different PLs belonging to the same class to be similar due to the absence of competition for ionization with PLs of different classes and due to the almost-identical composition of the mobile phase in which they are eluted under HILIC conditions [28]. Conceivably, the comparison between ESI(+)-FTMS spectra obtained for PC of different seafood products, reported in Figure S1, also enabled an evaluation of similarities or differences between seafood products from a quantitative point of view. In particular, the PC spectral profiles obtained for the two types of oysters were very similar, with prevalence of diacylic species with compositions 36:5 and 38:6, followed by those with compositions 40:6 and 40:7. Clams exhibited a spectral profile for PC that was more similar to those obtained for octopus (both mantle and tentacles), with PC(38:6) still representing the leading species, but other PCs, such as those with compositions o-38:6/p-38:5 and 34:1, became more relevant, and PC(36:5) showed a lower incidence compared to oysters. Finally, the PC spectral profile of shrimps was peculiar, with PC having 34:1 and 32:1 compositions becoming major species. Based on the PC distribution similarities, the analyzed products could thus be divided into three general groups: (i) oysters (both species), (ii) clams and octopuses, and (iii) shrimps. Not surprisingly, this grouping was consistent with that enabled by a comparison of the chromatographic profiles obtained for PC (see Figure 1).

In the case of each lysoPC, the composition of the single acyl or alkyl chain located on the molecular structure was easily inferred from a search on the LipidMaps database based on the accurate *m*/*z* ratio of the corresponding positive ion. The compositions of LPC detected in the lipid extracts of the analyzed products are reported in Figure 2 and Figure S2. Interestingly, the 16:0 chain was always prevailing, generally followed—in the case of oysters—by 18:0/18:1 and 20:1 chains and by the o-16:0 (in this case, no doubt exists on the chain identity due to the lack of C=C bonds) in the case of clams and octopuses (see Figure S2). In fact, up to three plasmanyl LPC were found in octopuses lipid extracts, whereas the LPC profile of shrimps was rather different from all the others, with the contemporary presence of several plasmanyl PCs (o-14:0/o:15/o-16:0/o-18:0) and of the acylic LPC with 18:1 and 18:0 chain detected in oysters. The occurrence of LPCs with saturated or monounsaturated residual acyl or alkyl chains—despite the presence of many PCs with a high number of C=C bonds—suggested that PC hydrolysis occurring naturally in seafood products mainly involves the chain located on the sn_2_ position of glycerol. Indeed, as emphasized by our recent study on mussels [17], most PCs of marine organisms are expected to bear polyunsaturated chains on the sn_2_ position.

The composition profiles of the PE class were quite different from those obtained for PCs; indeed, as emphasized in Figure S3 and by analogy with the results obtained for mussels [17], the PE class was dominated by plasmanyl/plasmenyl species and only a few diacylic species (with 36, 38, or 40 carbon atoms) were detected, although two of the latter (38:6 and 40:7) were the prevailing PEs in the case of shrimps. Interestingly, no saturated PE was detected—at least among signals exceeding the 5% abundance—and the number of C=C bonds could reach even 10 in the case of a PE detected in shrimp lipid extracts (40:10). Starting from this scenario, the prevalence of plasmanyl and plasmenyl species among LPE, inferred from Figure S4, was not surprising; yet, an acyl LPE—the 22:6 one—was found to be relevant in the case of clams, shrimps, and octopuses (especially in the mantle). A comparison with the PE profiles (see Figure S3) of these products suggested LPE(22:6) to be the hydrolysis byproduct of PEs with 38 or 40 carbon atoms, i.e., of PE in which the 22:6 chain was combined with a 16:0, 18:0, or 18:1 chain.

### 2.2. Effects of Thermal Treatments on Seafood Products: Evolution of LPE and LPC Species

**Figure 3:**
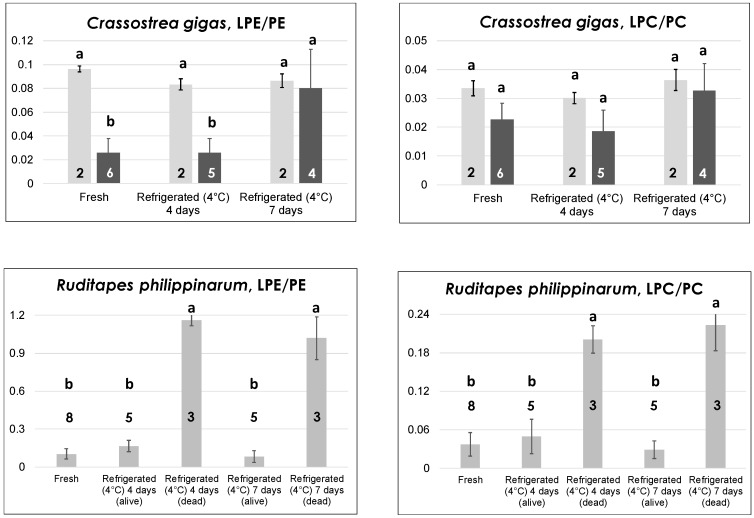
Comparison between lysophosphatidylethanolamines/phosphatidylethanolamines (LPE/PE) and LPC/PC ratios of ESI(+)-FTMS responses obtained from the HILIC–ESI(+)-FTMS analysis of lipid extracts of Japanese oysters (*C. gigas*) and Manila clams (*R. philippinarum*) that were subjected to lipid extraction soon after purchase (as fresh product) or after refrigeration at 4 °C for four or seven days in a laboratory refrigerator. Lighter bars in the graphs related to *C. gigas* represent data obtained for two lots of fresh oysters purchased in July, displaying anomalously high LPE/PE ratios (see text for an explanation for this finding). Mean values and standard deviations (indicated as error bars) reported relate to the number of replicates indicated for each type of sample. The results of a Tukey–Kramer test are reported in the form of sample type grouping, emphasized by letters. See text for details on the calculation of the LPE/PE and LPC/PC ratios from HILIC–ESI(+)-FTMS XIC chromatograms.

**Figure 4:**
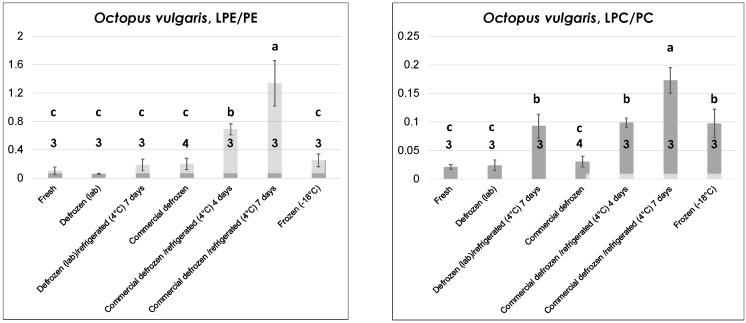
Comparison between LPE/PE and LPC/PC ratios of ESI(+)-FTMS responses obtained from the HILIC–ESI(+)-FTMS analysis of lipid extracts of common octopuses (*O. vulgaris*) that were subjected to lipid extraction soon after purchase or capture (fresh), as defrozen (either in lab or commercially), frozen or defrozen/refrigerated product. Mean values and standard deviations (error bars) that are plotted relate to the number of replicates indicated for each type of sample. The results of a Tukey–Kramer test are reported in the form of sample-type grouping and emphasized by letters. See text for details on the calculation of the LPE/PE and LPC/PC ratios from XIC chromatograms and on the different thermal treatments.

**Figure 5:**
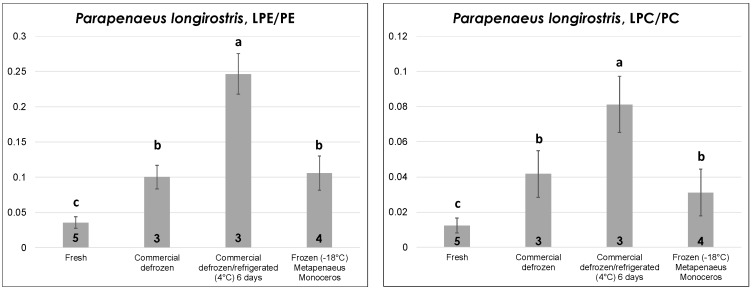
Comparison between LPE/PE and LPC/PC ratios of ESI(+)-FTMS responses obtained from the HILIC–ESI(+)-FTMS analysis of lipid extracts of (i) Mediterranean pink shrimps (*P. longirostris*) subjected to lipid extraction and analyzed as fresh or defrozen product or as defrozen product subjected to six days refrigeration at 4 °C; (ii) brown/speckled shrimps (*M. monoceros*) purchased as frozen product and thawed before lipid extraction and analysis. Mean values and standard deviations (indicated as error bars) reported relate to the number of replicates indicated for each type of sample. The results of a Tukey–Kramer test are reported in the form of sample type grouping, emphasized by letters. See text for details on the calculation of the LPE/PE and LPC/PC ratios from XIC chromatograms and on the different thermal treatments.

By analogy with one of our previous studies concerning mussels [18], the incidence of LPE and LPC—compared to their precursors, PE and PC—was exploited to evaluate if low temperature treatments, typically performed on the seafood products under study, could leave a mark on their PL profiles. For this aim, extracted ion current (XIC) chromatograms related to the four PL classes were first retrieved—both for fresh and for thermally treated products—by extracting from the HILIC–ESI(+)-FTMS TIC traces of the corresponding lipid extracts ion currents related to *m*/*z* intervals including all the species detected for each class, i.e., the *m*/*z* ranges of spectra shown in Figure 2 and Figures S1–S4. Afterwards, bands appearing in XIC traces in time intervals corresponding to those marked for PE, LPE, PC, and LPC in Figure 1 were integrated, respectively, and the resulting areas were adopted as estimates of the overall ESI(+)-FTMS responses for each class. This assumption is reasonable as no interference due to PLs of other classes was observed in ESI-FTMS spectra like those shown in Figure 2 and Figures S1–S4. Finally, XIC band areas were used to calculate LPE/PE and LPC/PC response ratios. The mean values of these for different seafood products and different thermal treatments are reported in Figure 3, Figure 4 and Figure 5 and in Figure S5 of the Supplementary Materials, accompanied by error bars representing standard deviations and numbers of samples analyzed for each treatment. In each case, the Tukey–Kramer statistical test was applied to evaluate if significant differences (at a 95% confidence level) occurred between different types of treatments for the same product. Thus, the treatments could be grouped according to the literal annotation shown in the figures. Notably, ratios reported in Figure 3, Figure 4 and Figure 5 and Figure S5 could not be influenced by between-run signal fluctuations, which often occur when LC-ESI-MS is performed, because they arose from responses obtained during the same run. This feature could be easily appreciated by considering the usually low relative standard deviation observed for a specific ratio in a particular product/treatment despite the fact that replicated lipid extractions and analyses related to the same seafood product and thermal treatment were performed over a relatively long time range. It can thus be hypothesized that the more pronounced variabilities—often observed for LPL/PL ratios related to particularly severe thermal treatments—were not due to analytical fluctuations but due to the higher variability in the individual reaction to those treatments (vide infra).

Since thermal treatments were often diverse for different seafood products, data reported in Figure 3, Figure 4 and Figure 5 and Figure S5 will be described and discussed in three subsections. The first subsection will focus on the three types of bivalve mollusks under analysis as the same treatment (refrigeration at 4 °C) was performed on them. The second and third subsections will be dedicated to octopuses and shrimps, respectively.

#### 2.2.1. Bivalve Molluscs: Oysters and Clams

In contrast to mussels, which can also be found on the market as glazed-frozen or defrozen products, oysters and clams are more valuable bivalves from a commercial point of view and are usually sold only as fresh (live) products. This feature sets severe transportation and storage constrains (see, for example, European Regulation 853/2004, Annexes VII and VIII), implying a constant refrigeration at a few °C (up to 4–6). For this reason, refrigeration at 4 °C (in a dry and clean container of a dedicated laboratory refrigerator) was applied to oysters and clams during our study. The process was initially prolonged for four days, a reasonable estimate of the maximum time that should be adopted for the storage of live bivalves at retail stores level. It was then purposely extended to seven days to simulate the effects of an inappropriate prolonged storage.

As shown in Figure S5 of the Supplementary Materials, prolonged refrigeration had a limited effect on oysters of *O. edulis* sp., i.e., the European flat oyster, since both the LPE/PE and the LPC/PC ratios were slightly increased with refrigeration time, but the variabilities observed for mean values after four and seven days (especially for LPC/PC ratios) were high enough to make the increase not statistically significant. As anticipated before, the increase in variability can be interpreted as the different response of individual oysters to the same thermal treatment, a phenomenon that was also observed for the other analyzed bivalve mollusks (vide infra). Nevertheless, the limited extent of PE and PC hydrolysis occurring during refrigeration, inferred from data of Figure S5, is consistent with previous studies dedicated to the low temperature tolerance of oysters of *O. edulis* sp. [29,30]. These oysters were reported to survive for at least 11 weeks (even without external feeding like in our study) in seawater at 3 °C [29]. 

A different scenario was observed for the other oyster species studied in the present work—*Crassostrea gigas*, i.e., the Japanese or Pacific oyster. Indeed, as shown by the darker bars in Figure 3, while the LPC/PC ratio exhibited a behavior like that observed for *O. edulis*, the LPE/PE ratio underwent a significant increase when passing from four to seven days of refrigeration at 4 °C. This result is in accordance with the generally lower capacity of *C. gigas* to survive at low temperatures, with high mortalities (>95%) reported after 3–7 weeks at 3 °C [29]. Interestingly, the sensitivity to thermal stress was greater for the LPE/PE ratio, in accordance with results previously obtained for mussels [18]. The data represented by light grey bars in the two graphs dedicated to *C. gigas* in Figure 3 deserve specific discussion. They are related to oysters of this species belonging to two lots purchased in the month of July, characterized by LPE/PE ratios significantly higher than those observed for fresh oysters purchased in other months. As apparent, both the LPE/PE and the LPC/PC ratio seemed to be unresponsive to prolonged refrigeration for these oysters, probably because an anomalously pronounced PE and PC hydrolysis had already occurred during storage in the fishery shop before purchase. Note that both *C. gigas* (originating from France) and *O. edulis* (originating from Greece or Italy) oysters analyzed in the present work were stored either in a seawater-filled (not refrigerated container) or in a dry container put in a refrigerating bench at the moment of purchase. Not surprisingly, anomalous LPE/PE values were observed for fresh *C. gigas* oysters stored in seawater purchased in July, the month that is usually characterized by the highest yearly temperatures. By analogy with data previously observed for mussels exposed to relatively high temperatures [18], the effect could be due to the combination of two factors: (i) the relatively high temperature reached by the seawater in which those oysters were stored (instead of being kept dry and into a refrigerated bench as prescribed by the current European legislation); and (ii) the increase in seawater temperature already at the level of the aquaculture plant. The result, which showed how sensitive to environmental conditions LPL/PL ratios of bivalve mollusks can be, was likely emphasized by the low capacity of *C. gigas* oysters to adapt to increasing temperatures [31].

The evolution of both LPL/PL ratios in the case of clams of *R. philippinarum* sp. (Manila clams) evidenced the presence of a more marked interindividual variability in the reaction to low temperature treatments compared to oysters. As shown in the bottom panels of Figure 3, low LPE/PE and LPC/PC ratios were always observed—in eight different lots—for fresh clams. However, refrigeration at 4 °C led to a surprising outcome. In three of the five lots that were subjected to refrigeration, a partial mortality already occurred after four days, as evidenced by the irreversible opening of valves for dead clams. The latter were thus separated from still-alive ones and lipid extraction was carried out separately. Then, HILIC–ESI(+)-FTMS analysis was performed on the lipid extracts arising from the two subgroups. As a result, both LPL/PL ratios were remarkably increased in dead clams, in strict analogy with results observed on mussels refrigerated at 4 °C [18]. Not surprisingly, a higher proportion of clams belonging to the three lots already displaying mortality after four days of refrigeration (but still alive after that time) were found dead when refrigerated for seven consecutive days at 4 °C. Nonetheless, their LPL/PL ratios were comparable to those of clams that were already dead after four days, whereas the few clams that were still alive after seven days of refrigeration exhibited ratios comparable to those of fresh ones (see Figure 3). It is worth noting that all clam samples examined in this work were stored in a seawater-filled container at room temperature, not in a refrigerating bench, at the moment of purchase. Once again, the three lots interested by a partial premature death were purchased in summer (July or early September), whereas the remaining two lots involved in prolonged refrigeration, which did not evidence any mortality even after seven days of refrigeration, were acquired in spring. Consequently, although all clams appeared comparable in terms of LPL/PL ratios when purchased, the more pronounced thermal stress expected for those purchased in hotter months due to the lack of a correct storage appeared to influence their subsequent behavior upon refrigeration. Apart from the discovery of such subtle effects, the monitoring of LPL/PL ratios in the case of clams could be very useful to preserve consumer safety and unveil inappropriate commercial conducts if the target product was represented by deshelled clams, sometimes available in fishery shops (like mussels). Indeed, clams dead before being deshelled due to a too-long or inappropriate storage would be easily recognized from those still alive before their valves were removed.

#### 2.2.2. Octopuses

As emphasized by graphs of LPE/PE and LPC/PC ratios in Figure 4, a higher number of thermal treatments had to be considered in the case of octopuses (all of *O. vulgaris* sp.), to account for the greater variety of thermal histories expected for this seafood product compared to bivalve mollusks. Tentacles were considered for lipid extraction and analysis, although, as discussed before and as shown in Figures S1–S4, the profiles of the four PL classes of interest were similar for the octopus’s mantle, which can also be used as edible part, once internal organs are removed. Two octopuses purchased in local fishery shops (where they were properly stored in a refrigerated bench) and one captured along the coasts of Apulia, 20 km north of Bari, and kept at 4 °C for ca. 24 h before being transferred to the laboratory were considered as fresh products. After the sampling of one tentacle for lipid extraction, each of the three octopuses was purposely frozen at −18 °C. Then, after thawing at 4 °C, a new tentacle was sampled and the lipid extraction/LC-MS analysis sequence was performed in each case. The resulting samples were considered as arising from “lab-made” defrozen octopuses. The octopus samples set was completed by defrozen and frozen (vacuum-sealed) *O. vulgaris* octopuses obtained commercially and by lab-made or commercially defrozen octopuses purposely stored for up to seven days in the lab refrigerator at 4 °C.

Data reported in Figure 4 indicate peculiar differences in the evolution of the LPE/PE and the LPC/PC ratios. Indeed, both were low and comparable in the case of fresh octopuses and defrozen ones (both lab-made and commercial) that were subjected to lipid extraction and analysis soon after preparation and without any further refrigeration. On the other hand, only the LPC/PC ratio was significantly increased in commercial frozen octopuses (compared to fresh or defrozen ones). This feature is very interesting as it suggests the possibility of distinguishing fresh octopuses from those claimed as fresh but obtained after thawing of frozen products, which represents a typical fraud. Lab-made defrozen octopuses stored for seven days at 4 °C (i.e., for an inappropriate time range) exhibited a significant increase of the LPC/PC ratio, whereas both LPL/PL ratios appeared sensitive to progressive refrigeration of commercially defrozen octopuses, with a steady increase occurring after four and seven days of refrigeration (see Figure 4). Based on these considerations, octopuses represented the only product, among those under consideration in this study, for which the LPC/PC ratio provided more refined information than the LPE/PE in terms of distinction between different thermal treatments.

#### 2.2.3. Shrimps

The edible part (i.e., the deshelled body) of fresh and commercial defrozen shrimps of *P. longirostris* sp. (pink shrimp)—one of the leading shrimp species from a commercial point of view in the Mediterranean area—was subjected to lipid extraction and analysis during this study after purchase from local fisheries. Unfortunately, frozen shrimps of that species were not available locally; thus—for the sake of comparison—peeled, vacuum-sealed frozen shrimps of *Metapenaeus monoceros* sp. (speckled/brown shrimp), which are native of Indian or Pacific Oceans and are intensively imported in Europe as frozen product, were examined. As shown in Figure 5, the LPE/PE and LPC/PC ratios had a very similar trend with thermal history, with fresh shrimps displaying the lowest values, as expected. In contrast to octopuses, defrozen shrimps displayed significantly higher LPL/PL ratios than fresh ones, thus indicating a subtler correlation of this parameter to the thermal history in the case of shrimps. As observed before for fresh bivalves and for defrozen octopuses, prolonged refrigeration (up to six days) at 4 °C led to a significant increase in the LPL/PL ratio. Finally, frozen shrimps displayed LPL/PL ratios statistically comparable to those obtained for defrozen ones, which is reasonable given both products experienced a freezing/thawing sequence before lipid extraction and analysis.

## 3. Discussion

Data described above emphasize the complexity related to the thermal histories of the investigated seafood products. Nonetheless, some general trends emerged from them and were often consistent with those observed for mussels in a previous study [18]. Indeed, except for *O. edulis*—an oyster species inherently capable of resisting better and for longer times to the exposure to low temperatures—the significant increase in the relative incidence of lysoPLs, like LPC and LPE, appeared as a marker of the prolonged refrigeration of bivalve mollusks, ultimately leading to their death. Additionally, that increase also occurred when bivalves were stored in the retail shop into nonrefrigerated, seawater-filled vessels that were not equipped with a water recirculation system, especially when the ambient temperature was likely higher than 25 °C, thus leading to a rapid heating of the storage water. Both these results were in excellent accordance with those previously found for mussels [18]. They emphasized how HILIC–ESI-MS, even if performed with mass spectrometers with lower performances than those adopted during this study (high resolution/accuracy are not mandatory if overall PL class responses are searched for), can easily unveil improper, or even fraudulent, practices relating to the commercialization of seafood products. The increase in LPLs occurring in mussels after different kinds of thermal stress was previously attributed to the action of endogenous phospholipases [18], which occurs in vivo when live mollusks are involved, like in the present case for oysters and clams. A careful evaluation of ESI(+)-FTMS spectra obtained for LPC and LPE in thermally stressed bivalves during this study indicated that the process has a certain degree of specificity. Indeed, as shown in Figures S6 and S7 of the Supplementary Materials, several polyunsaturated species—including those including the well-known 22:6, 20:5, and 20:4 acyl chains—emerged in the profile of LPC for oysters and clams refrigerated for seven days. The most evident effect concerned LPC (22:6), which was virtually undetected in fresh clams and became the leading species in clams dead after seven days of refrigeration (see Figures S2 and S6). Surprisingly, LPE (22:6) showed an entirely reversed trend by almost disappearing in the LPE profile of dead clams, whereas it was the leading species of the class in live clams (see Figures S4 and S7). Since polyunsaturated acyl chains are expected to be mainly located in the sn_2_ position of glycerol in bivalves [17], these results would imply a significant role of phospholipases with sn_1_-specificity in the case of PC and those with sn_2_-specificity in the case of PE during low temperature stresses on bivalves. Results consistent with this hypothesis were also found for octopuses and shrimps. In fact, as evidenced in Figure S6, polyunsaturated LPC with 20 or 22 carbon atoms on the acyl chain were clearly increased, while the corresponding LPE species were less abundant in the lipid extracts of commercial octopuses and shrimps purchased as defrozen products and then subjected to prolonged refrigeration. As for bivalves, the occurrence of this process, which is not compliant with correct storage procedures for defrozen seafood, could be easily traced back by monitoring the LPL/PL ratios (see Figure 4 and Figure 5). Moreover, the ratios appeared able to unveil further putative frauds, such as selling a frozen/thawed octopus as a fresh one or defrozen shrimps as fresh ones, due to the presence of significant differences in their values in both types of product.

On the basis of these considerations, HILIC–ESI-MS, which has already been applied with success in several different lipidomics-oriented contexts, emerge as a very promising candidate among MS-based analytical approaches that could help in controlling product safety and quality. The same approach could also be useful in unveiling frauds, the incidence of which is expected to increase with the rapid increase in seafood consumption worldwide in the future. 

## 4. Materials and Methods 

### 4.1. Chemicals

LC-MS grade water, methanol and acetonitrile, HPLC-grade chloroform, and analytical-grade ammonium acetate and sodium chloride were all purchased from Sigma-Aldrich (Milan, Italy).

### 4.2. Seafood Products Collection, Homogenization, and Lipid Extraction

Seafood products analyzed during the present work were all purchased from local commercial sources, with the exception of one octopus that was caught along the Adriatic Sea coast, 20 km north of Bari (Apulia region, Italy). The lots relating to each product were purposely acquired in different months of the year in order to evaluate if different climatic conditions could influence the incidence of lysoPLs in fresh products and, eventually, in thermally treated ones. As discussed before in the paper, this was actually the case for oysters of *Crassostrea gigas* sp. and for clams of *Ruditapes philippinarum* sp. that were purchased in summer months like July and September.

A brief description of the type and storage conditions (adopted in the fishery shop or supermarket) of seafood products investigated here is reported in the following list:

(1) farmed live oysters of *C. gigas* sp. (origin: France) and *O. edulis* (origin: Greece or Italy), stored either in seawater in nonrefrigerated containers or dry, in refrigerated benches;
(2) farmed live clams of *R. philippinarum* sp. (origin: Italy) stored in seawater in nonrefrigerated containers;
(3) fresh octopuses of *O. vulgaris* sp. (origin: Italy), refrigerated at 0–4 °C;
(4) defrozen octopuses of *O. vulgaris* sp. (origin: Italy), stored under refrigeration at 0–4 °C;
(5) frozen and vacuum-sealed octopuses of *O. vulgaris* sp. (origin: Italy), stored at −18 °C;
(6) fresh shrimps of *P. longirostris* sp. (origin: Italy), stored under refrigeration at 0–4 °C;
(7) frozen and vacuum-sealed shrimps of *M. monoceros* sp. (origin: Indian Ocean), stored at −18 °C.

Except for the octopus caught locally, which had to be stored for ca. 24 h at 4 °C for practical reasons before being transferred to the laboratory, all samples were transferred to the laboratory within 30 min from purchase. Each of the lots of oysters, clams, and shrimps was divided into sublots—one of the latter was soon subjected to homogenization, lipid extraction, and LC-MS analysis; the others were subjected to different thermal treatments (described in detail in the Results section) before lipid extraction and analysis. In the case of octopuses, each individual represented a single lot, thus a small piece was withdrawn using a knife (from the mantle or from a tentacle) soon after transfer to the laboratory for immediate homogenization, lipid extraction, and analysis. The remaining product, as a whole, was subjected to a thermal treatment before proceeding with a further sampling, lipid extraction, and analysis.

In the case of species characterized by softer tissues, among those under study—bivalve mollusks and shrimps (intending the edible part of the body, in the latter case)—homogenization was performed into a 8 mL Potter–Elvehjem homogenizer equipped with a teflon pestle, which was placed into an ice bath and contained 2 mL of LC-MS grade methanol, previously refrigerated at 4 °C. The preliminary addition of cold methanol was aimed at minimizing hydrolytic effects occurring during homogenization and related to endogenous phospholipases [18]. In the case of clams and shrimps, two individuals could be easily cohomogenized after the removal of the valves and of the head and exoskeleton, respectively. In the case of oysters, due to the remarkable dimensions of the edible part (with a weight ranging from 10 to 27 g per individual), only one individual could be subjected to homogenization and lipid extraction at a time. In the case of octopuses, due to the significantly greater hardness of the tissues of mantle and tentacles, the dissected part had to be cut into smaller pieces before starting manual homogenization. Dissection was performed with a knife while the original octopus sample was totally immersed into cold methanol and the solvent container was placed into an ice bath in order to minimize the action of phospholipases that could alter the observed phospholipid profiles. The resulting suspension was transferred into a mortar that already contained 2 mL of LC-MS grade methanol and homogenized manually using a Teflon pestle. Frozen products, such as octopuses or shrimps, were thawed by overnight storage in a refrigerator at 4 °C before being subjected to homogenization. 

In all cases, seafood products homogenates were subjected to lipid extraction according to the Bligh and Dyer protocol [32], which had been previously performed successfully on mussels in our laboratory [17,18]. In particular, excess liquid, including methanol and eventual water released by the products, was preliminarily removed from the homogenization device using a Pasteur pipette. Then, 1 g of the homogenate was transferred into a beaker and 1 and 2 mL of cold HPLC-grade chloroform and LC-MS grade methanol, respectively, were added. After 5 min of agitation on a magnetic stirrer, the homogenate/solvents mixture was subjected to centrifugation at 5200× *g* for 5 min. The surnatant was then separated, and 1 mL of chloroform and the same volume of a 1% NaCl solution—prepared with LC-MS water—were added. After appropriate stirring and subsequent decantation, a separation was observed between the opaque water/methanol phase and the limpid chloroform phase. Subsequent centrifugation at 5200× *g* for 5 min led to clarification of the water/methanol phase and creation of a dense, whitish interface between it and the bottom, chloroform-rich phase. The latter, including lipids, was separated carefully and was often slightly colored due to the coextraction of natural pigments contained in the tissues/organs of some of the analyzed seafood products. Specifically, clam extracts had a light orange color; oyster extracts were sometimes colorless and in other cases, yellowish. Shrimp extracts were pinkish, while octopus extracts were colorless, since the chromatophores of these mimetic organisms consists of hydrophilic pigments dispersed into specific cells. All the chloroform extracts were transferred into glass vials equipped with screw caps having a chloroform-resistant internal membrane; the vial headspace was saturated with nitrogen before closing in order to minimize lipid oxidation processes eventually occurring before analysis. The vials were then stored at −20 °C before proceeding to LC-MS analysis, which usually occurred within 6 h from the extraction of lipids. A dilution was usually necessary before sample injection to ensure optimal chromatographic resolution and to avoid saturation of the ESI-MS signal. Most extracts were diluted 1:20 (*v*/*v*) in a methanol/chloroform 1:1 mixture; only octopus extracts were diluted 1:10 (*v*/*v*) in the same mixture, since the extraction process from the hard tissue of octopus was expected to be less effective than those occurring on the other products.

### 4.3. HILIC–ESI-FTMS Instrumentation and Operating Conditions

HILIC separations were performed using an Ultimate 3000 UHPLC system (Thermo Scientific, Waltham, MA, USA), equipped with a narrow-bore, high efficiency Ascentis Express HILIC column (150 × 2.1 mm ID, 2.7 μm particle size), preceded by an Ascentis Express HILIC security guard cartridge (5 × 2.1 mm ID), both packed with a silica stationary phase and manufactured by Supelco (Bellefonte, PA, USA). Five μL of preliminarily diluted lipid extracts were injected using the autosampler of the Ultimate 3000 system. The following binary elution program, based on methanol (solvent A) and acetonitrile (solvent B)—both containing 10% (*v*/*v*) of water and 1 mmol/L of ammonium acetate—was adopted for separations, performed at a 0.3 mL/min flow rate: 0–6 min, isocratic at 95% solvent B; 6–15 min, linear gradient from 95 to 65% solvent B; 15–25 min, isocratic at 65% solvent B; 25–30 min, return to the initial composition, followed by a 10 min equilibration time.

High resolution FTMS measurements were performed after HILIC separations by a Q-Exactive mass spectrometer (Thermo Scientific, Waltham, MA, USA), a hybrid mass spectrometer including a quadrupole connected through a so-called C-trap to an orbital trap (Orbitrap) and equipped with a heated electro spray ionization (HESI) source for LC-MS coupling. The main electrospray and ion optics parameters adopted during HILIC–FTMS acquisitions were the following: sheath gas flow rate, 35 (arbitrary units); auxiliary gas flow rate, 15 (arbitrary units); spray voltage, +3.5 kV (positive polarity); capillary temperature, 320 °C; S-Lens RF Level, 100 (arbitrary units). Positive ion MS spectra were acquired in the *m*/*z* range 120–1200 after setting a mass resolving power of 70,000 (measured at *m*/*z* 200), an Orbitrap fill-time of 200 ms, and automatic gain control (AGC) level at 5 × 10^5^. To ensure a mass accuracy always better than 1 ppm, the *m*/*z* scale of the Q-Exactive spectrometer was periodically calibrated using a solution containing caffeine, MRFA peptide, and a mixture of oligomeric fluorinated phosphazines (Ultramark), provided by Thermo Scientific. 

## Supplementary Materials

The following are available online, Figure S1: Comparison of typical ESI(+)-FTMS spectra obtained for PC in fresh seafood products, Figure S2: Comparison of typical ESI(+)-FTMS spectra obtained for LPC in fresh seafood products, Figure S3: Comparison of typical ESI(+)-FTMS spectra obtained for PE in fresh seafood products, Figure S4: Comparison of typical ESI(+)-FTMS spectra obtained for LPE in fresh seafood products, Figure S5: Comparison of LPE/PE and LPC/PC ESI(+)-FTMS response ratios obtained for fresh or refrigerated European flat oysters (*Ostrea Edulis*), Figure S6: Comparison of typical ESI(+)-FTMS spectra obtained for LPC in seafood products after thermal treatments leading to the maximum accumulation of LPC, Figure S7: Comparison of typical ESI(+)-FTMS spectra obtained for LPE in seafood products after thermal treatments leading to the maximum accumulation of LPE.
