# Supplementary material for: Tracing the Thermal History of Seafood Products through Lysophospholipid Analysis by Hydrophilic Interaction Liquid Chromatography–Electrospray Ionization Fourier Transform Mass Spectrometry
Source: Molecules. 2018 Aug 31;23(9):2212. doi: 10.3390/molecules23092212 (PMC6225239; doi:10.3390/molecules23092212)
Supplement: Supplementary file 1 [file molecules-23-02212-s001.pdf]

Supplementary Materials

Tracing the thermal history of seafood products through *lyso*-phospholipid analysis by hydrophilic interaction liquid chromatography-electrospray ionization Fourier-transform mass spectrometry

Ilario Losito[1,2,*], Laura Facchini[1], Rosa Catucci[1], Cosima Damiana Calvano[1,2], Tommaso R.I. Cataldi[1,2], Francesco Palmisano[1,2]

[1] Dipartimento di Chimica, Università degli Studi di Bari "Aldo Moro", Via E. Orabona 4, 70126 Bari, Italy; ilario.losito@uniba.it
[2] Centro Interdipartimentale SMART, Università degli Studi di Bari "Aldo Moro", Via E. Orabona 4, 70126 Bari, Italy
* Correspondence: Ilario.losito@uniba.it; Tel.: +39-080-544-2506

Titles of supplementary figures, as reported at the end of the main manuscript:

Figure S1. Comparison of typical ESI(+)-FTMS spectra obtained for PC in fresh seafood products

Figure S2. Comparison of typical ESI(+)-FTMS spectra obtained for LPC in fresh seafood products

Figure S3. Comparison of typical ESI(+)-FTMS spectra obtained for PE in fresh seafood products

Figure S4. Comparison of typical ESI(+)-FTMS spectra obtained for LPE in fresh seafood products

Figure S5. Comparison of LPE/PE and LPC/PC ESI(+)-FTMS response ratios obtained for fresh or refrigerated European flat oysters (*Ostrea Edulis*)

Figure S6. Comparison of typical ESI(+)-FTMS spectra obtained for LPC in seafood products after thermal treatments leading to the maximum accumulation of LPC

Figure S7. Comparison of typical ESI(+)-FTMS spectra obtained for LPE in seafood products after thermal treatments leading to the maximum accumulation of LPE

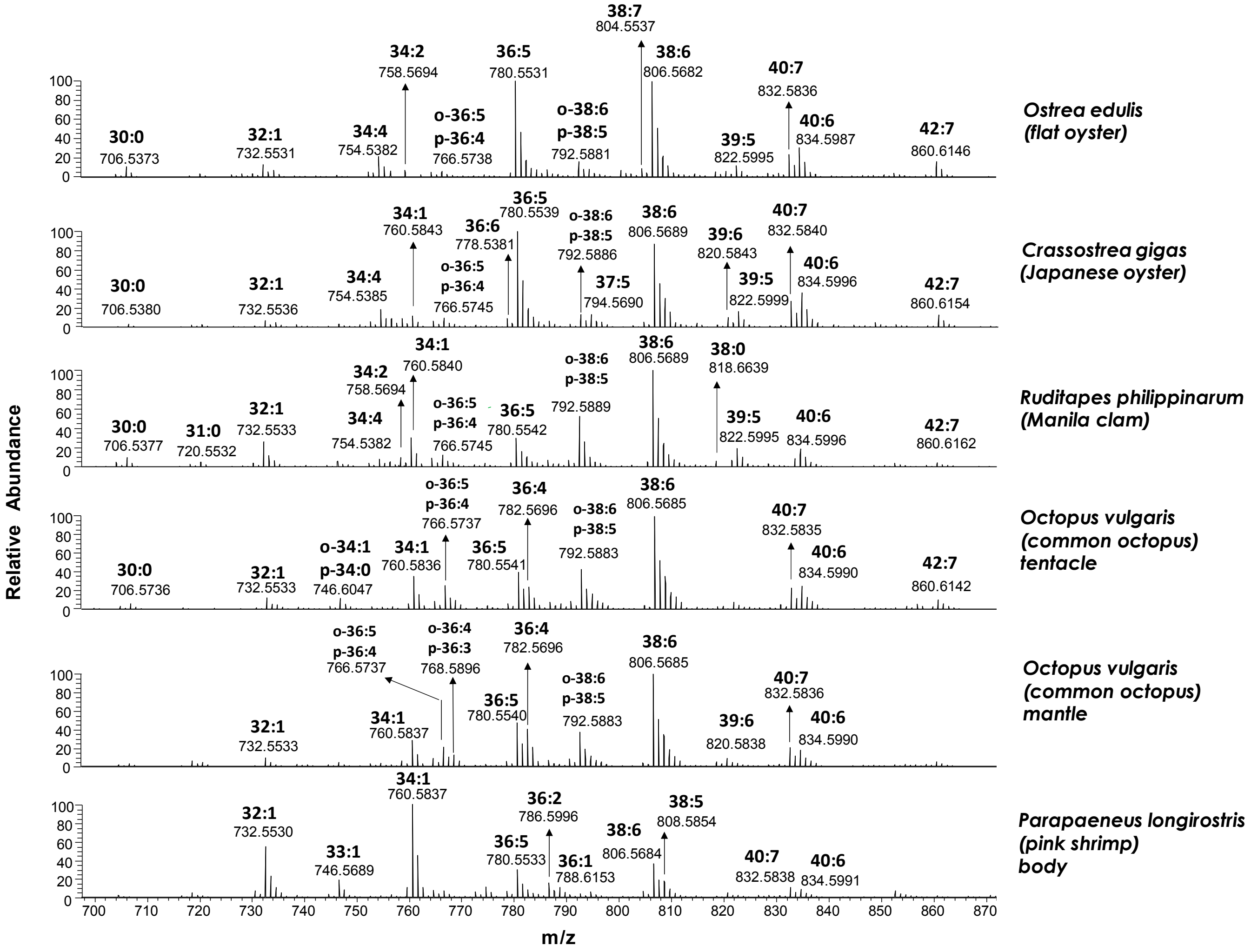

Figure S1. Comparison between typical ESI(+)-FTMS spectra obtained for the PC class during the HILIC-ESI(+)-FTMS analysis of lipid extracts obtained from different fresh seafood products. Spectra were averaged in the retention time interval 7.3 - 11.6 min (see Figure 1 in the paper).

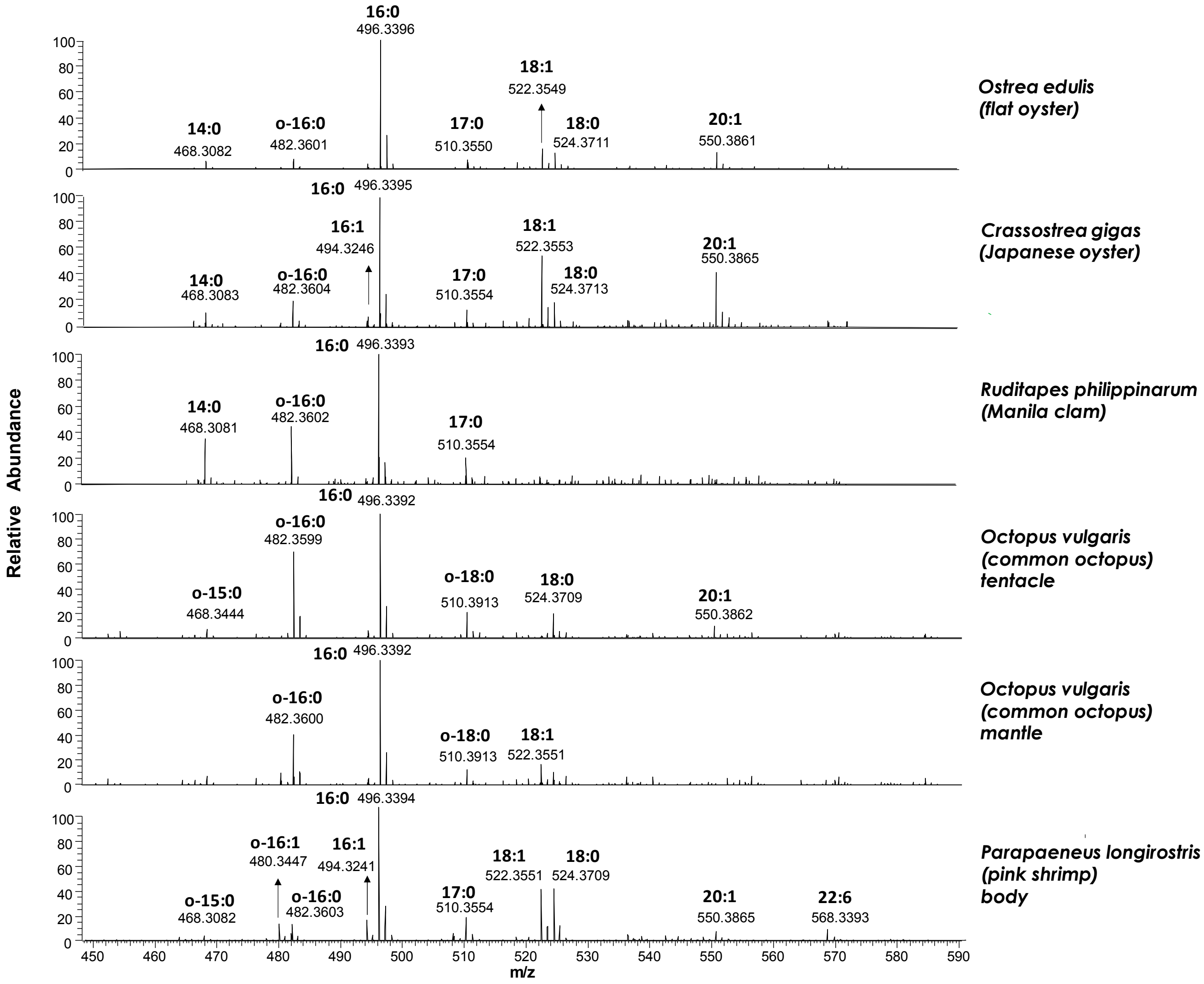

Figure S2. Comparison between typical ESI(+)-FTMS spectra obtained for the LPC class during the HILIC-ESI(+)-FTMS analysis of lipid extracts obtained from different fresh seafood products. Spectra were averaged in the retention time interval 14.6 - 16.7 min (see Figure 1 in the paper).

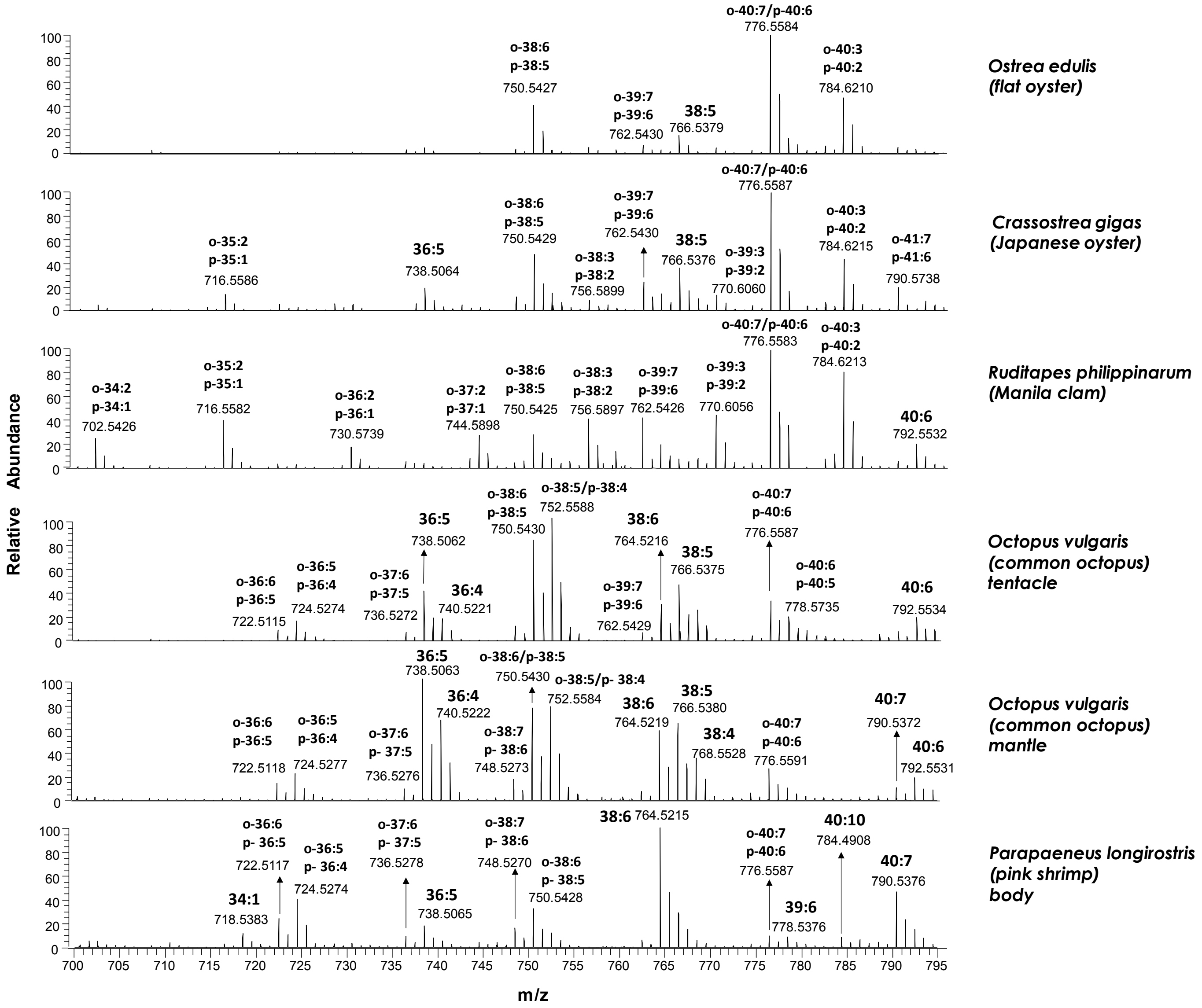

Figure S3. Comparison between typical ESI(+)-FTMS spectra obtained for the PE class during the HILIC-ESI(+)-FTMS analysis of lipid extracts obtained from different fresh seafood products. Spectra were averaged in the retention time interval 2.3 – 3.4 min (see Figure 1 in the paper).

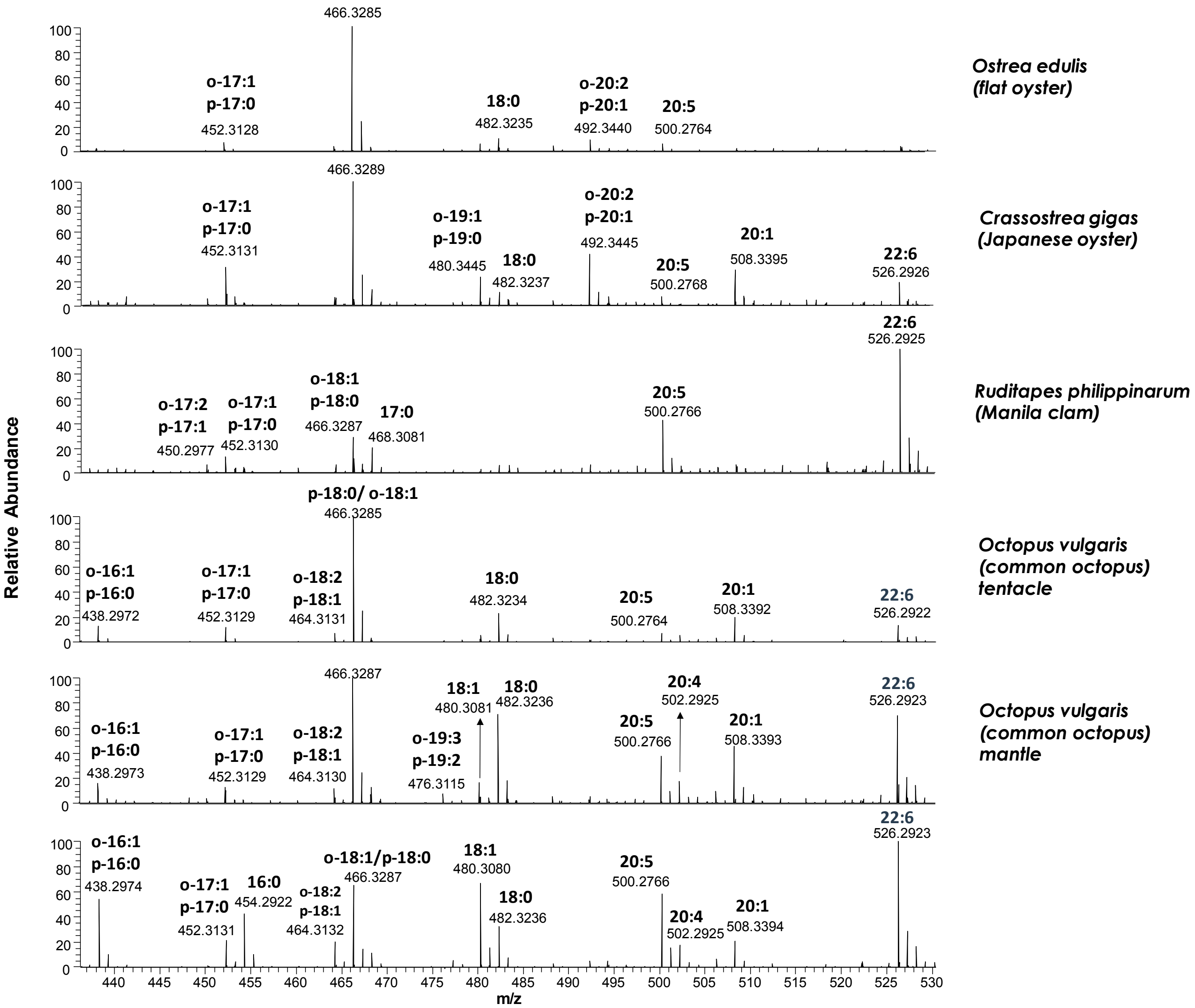

Figure S4. Comparison between typical ESI(+)-FTMS spectra obtained for the LPE class during the HILIC-ESI(+)-FTMS analysis of lipid extracts obtained from different fresh seafood products. Spectra were averaged in the retention time interval 3.4 – 4.3 min (see Figure 1 in the paper).

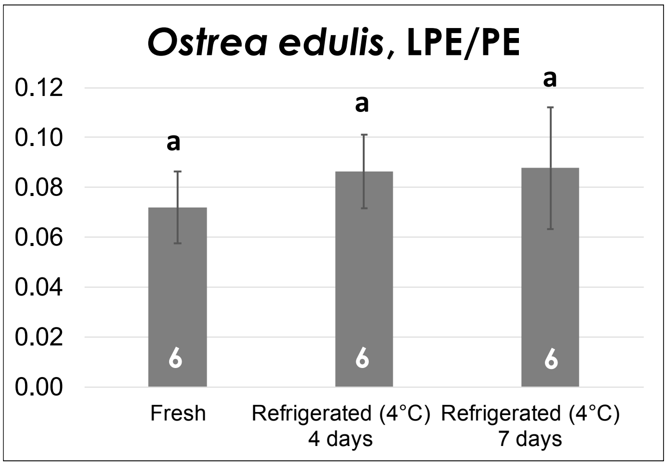

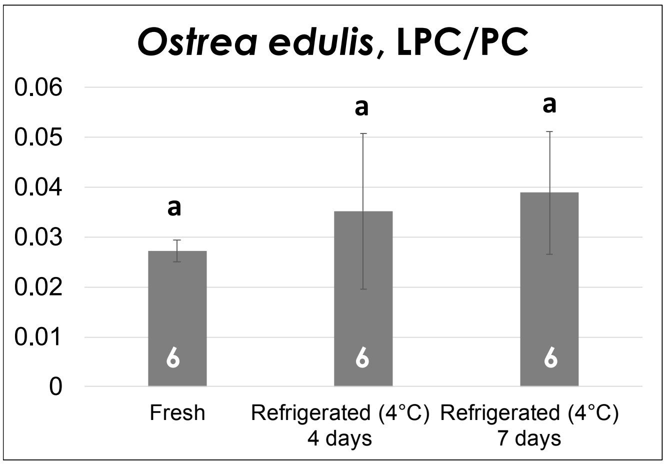

Figure S5. Comparison between LPE/PE and LPC/PC ratios of ESI-(+)-FTMS responses obtained from the HILIC-ESI(+)-FTMS analysis of lipid extracts of European flat oysters (*O. edulis*) subjected to lipid extraction soon after purchase (as fresh product) or after refrigeration at 4°C for 4 or 7 days in a laboratory refrigerator. Mean values and standard deviations (indicated as error bars) are reported, referred to the number of replicates indicated for each type of sample. The results of a Tukey-Kramer test are reported in the form of sample type grouping, emphasized by letters. See the main text for details on the calculation of the LPE/PE and LPC/PC ratios from XIC chromatograms.

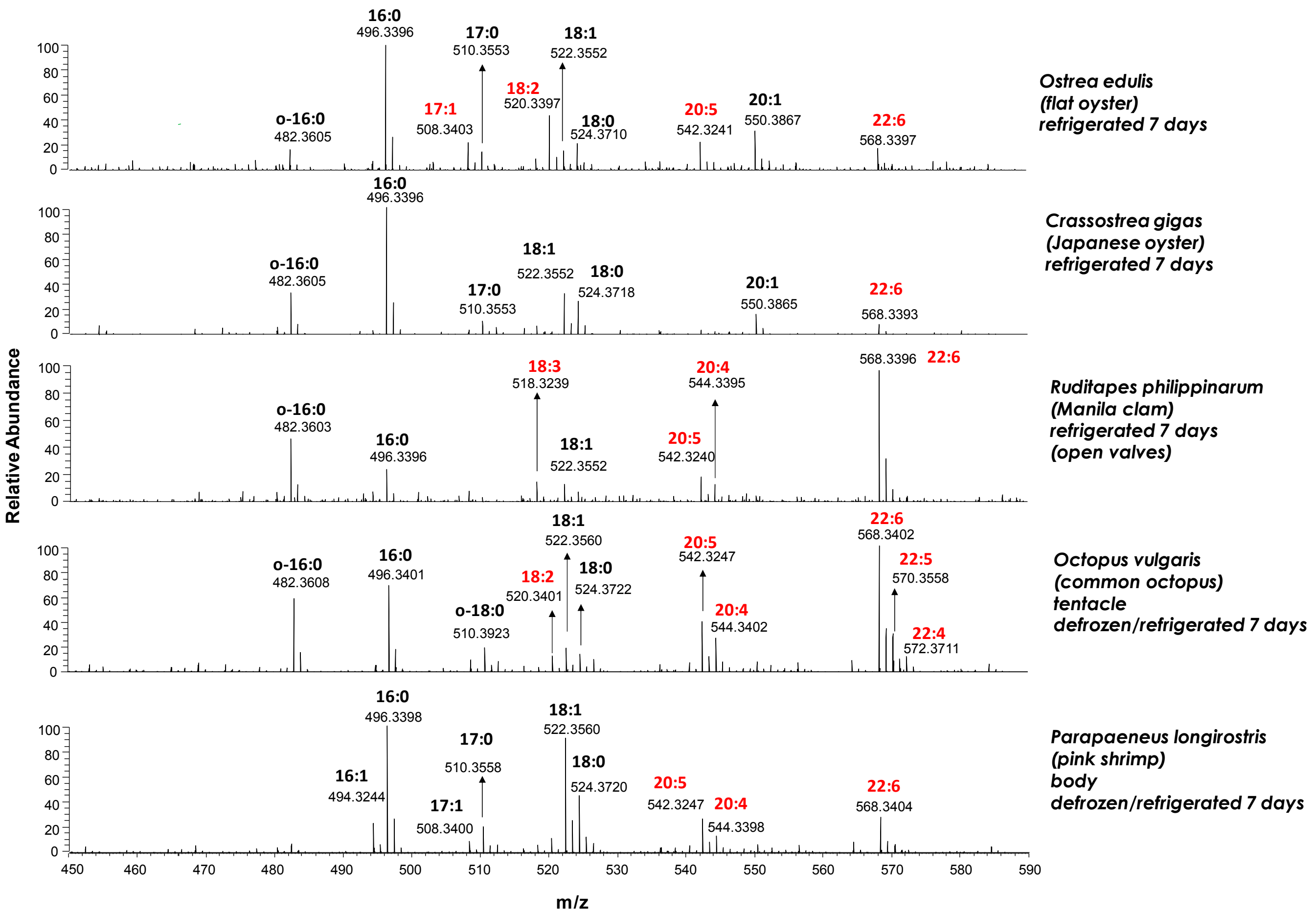

Figure S6. Comparison between ESI(+)-FTMS spectra obtained for the LPC class during the HILIC-ESI(+)-FTMS analysis of lipid extracts obtained from seafood products after thermal treatments leading to the maximum accumulation of LPC species. Red labels indicate LPC species that exhibited a significant variation (increase or decrease) in treated products with respect to fresh ones. Spectra were averaged in the retention time interval 14.6 - 16.7 min (see Figure 1 in the paper).

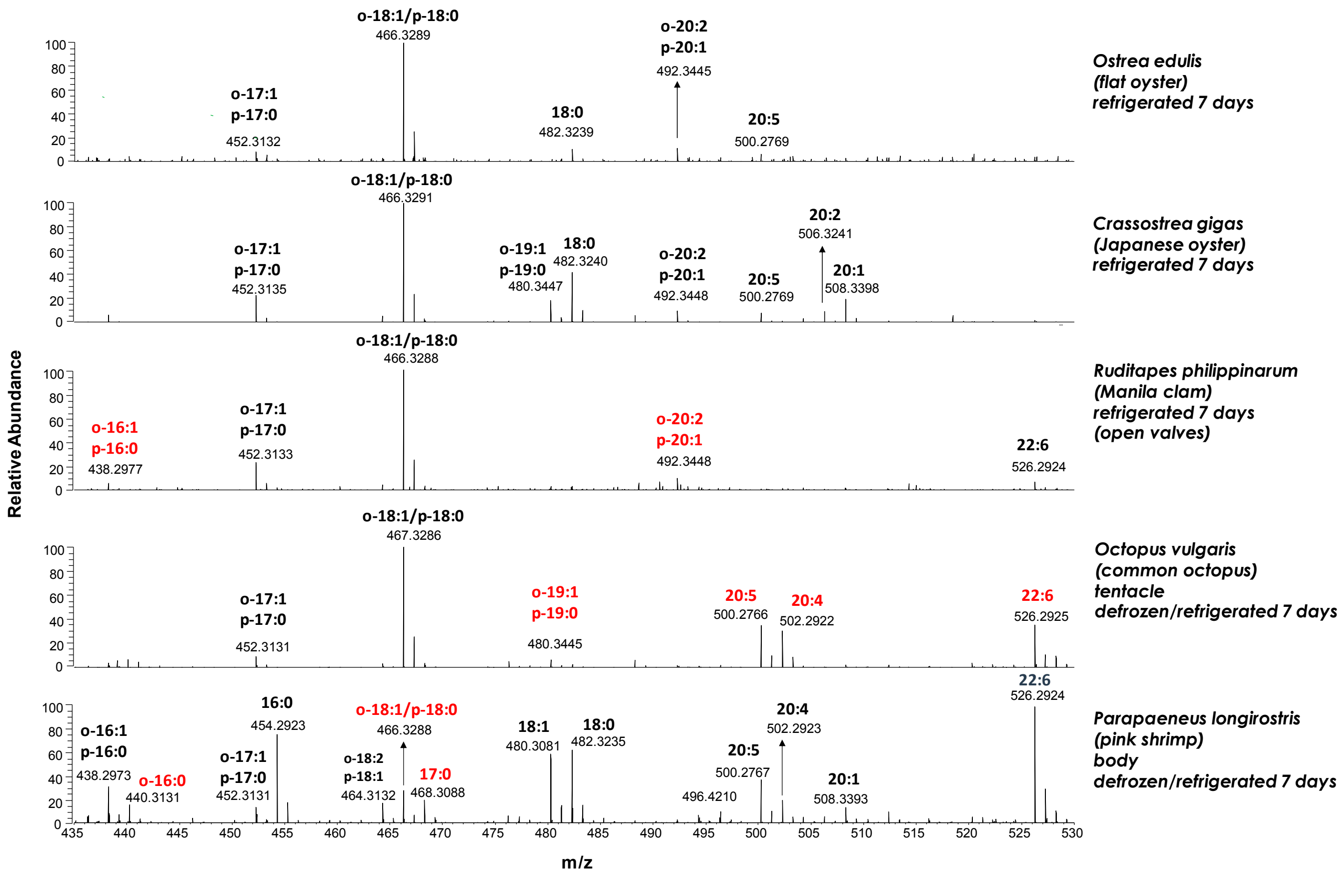

Figure S7. Comparison between ESI(+)-FTMS spectra obtained for the LPE class during the HILIC-ESI(+)-FTMS analysis of lipid extracts obtained from seafood products after thermal treatments leading to the maximum accumulation of LPE species. Red compositional labels indicate LPE species that exhibited a significant variation (increase or decrease) in treated products with respect to fresh ones. Spectra were averaged in the retention time interval 3.4 – 4.3 min (see Figure 1 in the paper).
